# Updating a dataset of labelled objects on raw video sequences with unique object IDs^✰^

**DOI:** 10.1016/j.dib.2022.107892

**Published:** 2022-02-02

**Authors:** Takehiro Tanaka, Hyomin Choi, Ivan V. Bajić

**Affiliations:** School of Engineering Science, Simon Fraser University, 8888 University Drive, Burnaby, BC V5A 1S6, Canada

**Keywords:** Object tracking, video coding, video compression

## Abstract

We present an update to the previously published dataset known as SFU-HW-Objects-v1. The new dataset is called SFU-HW-Tracks-v1 and contains object annotations with unique object identities (IDs) for the High Efficiency Video Coding (HEVC) v1 Common Test Conditions (CTC) sequences. For each video frame, ground truth annotations include object class ID, object ID, and bounding box location and its dimensions. The dataset can be used to evaluate object tracking performance on uncompressed video sequences and study the relationship between video compression and object tracking, which was not possible using SFU-HW-Objects-v1.

## Specifications Table

**Table:** 

Subject	Computer Science
Specific subject area	Computer Vision and Pattern Recognition
Type of data	Annotations (text files)
How the data were acquired	The annotated data was obtained by assigning a unique object ID to each object in the existing object detection dataset SFU-HW-Objects-v1. This was done by a semi-automated tracking followed by manual inspection and correction, as described in the article.
Data format	Analyzed
Description of data collection	Data was generated by applying correlation tracking to object detection labels in SFU-HW-Objects-v1, followed by manual correction of tracks. This resulted in unique object IDs, identifying the same object in multiple frames, which do not exist in the original dataset.
Data source location	Institution: Simon Fraser University City/Town/Region: Burnaby, British Columbia Country: Canada Latitude and longitude (and GPS coordinates, if possible) for collected samples/data: Latitude: 49.276765, Longitude: −122.917957 Primary data sources: Raw HEVC v1 CTC video sequences maintained by ITU-T JCT-VC: ftp://hevc@mpeg.tnt.uni-hannover.de/testsequences/ (For the regular use of the primary dataset, access details can be obtained from JCT-VC Chairs: https://www.itu.int/en/ITU-T/studygroups/2017–2020/16/Pages/video/jctvc.aspx)
Data accessibility	Repository name: Mendeley Direct URL to data: http://dx.doi.org/10.17632/d5cc83ks6c.1 Instructions for accessing these data: Secondary data, which this paper describes, is publicly available at the above URL. **Primary data** requires a password and is available at: ftp://hevc@mpeg.tnt.uni-hannover.de/testsequences/ For the regular use of the primary dataset, access details can be obtained from JCT-VC Chairs: https://www.itu.int/en/ITU-T/studygroups/2017–2020/16/Pages/video/jctvc.aspx

## Value of the Data

• New data include unique object IDs, which identify the same object in multiple frames in the uncompressed HEVC v1 CTC test sequences.
• The expanded dataset enables benchmarking of object trackers on uncompressed HEVC v1 CTC test sequences and can be used to study the relationship between video compression and object tracking.

## 1 Data Description

We prepared object tracking annotations for 13 high efficiency video coding (HEVC) v1 common test conditions (CTC) video sequences in the YUV420 format [2], as shown in Table 1. These sequences are uncompressed and can be acquired from joint collaborative team on video coding (JCT-VC). The new data extend the previously presented SFU-HW-Objects-v1 dataset, and the extended dataset is called **SFU-HW-Tracks-v1**. For each video frame, ground truth annotations include object class ID, object ID, and bounding box location and its dimensions. The dataset SFU-HW-Tracks-v1 has separate folders for each class of sequences (B, C, D, E), which differ in resolution, and each class folder contains individual sequence folders. Each sequence folder contains one annotation file per frame, which is a text file and can be viewed in any text editor. Each row in the annotation file corresponds to an object in the corresponding frame, and contains the following information: [Class ID, Object ID, x*,* y*,* w*,* h]. Class ID represents the identifier of an object class, for example “person,” “bicycle,” etc. All the class IDs are listed in Table 2, and they are all part of Common Objects in Context (COCO) object classes [3]. Object ID refers to the unique identity of each object. For example, if a frame contains two persons, unique IDs are provided for person 0 and person 1. Finally, x and y are the horizontal and vertical coordinates of the object's bounding box in relative coordinates (relative to the frame dimensions, as explained below), while w and h are the relative dimensions of the bounding box. The center position of object bounding box in relative coordinates is obtained from the absolute coordinates $x^{*}$ and $y^{*}$ (from the top-left corner), and frame width N and height M, as:

(1) $$x=\frac{x^{*}}{N}$$

(2) $$y=\frac{y^{*}}{M}$$

**Table 1:** List of ground truths prepared for HEVC v1 CTC sequences adapted from [1].

Class	Sequence name	Resolution	Frame count	Frame rate (Hz)	Bit depth	Class IDs	Number of object classes
B	BasketballDrive	1920**⋅**1080	500	50	8	[0, 32, 56]	**4**
B	Cactus	1920**⋅**1080	500	50	8	[58]	**1**
B	Kimono	1920**⋅**1080	240	24	8	[0, 26]	**2**
B	ParkScene	1920**⋅**1080	240	24	8	[0, 1, 13]	**4**
C	BasketballDrill	832**⋅**480	500	50	8	[0, 32, 56]	**4**
C	PartyScene	832**⋅**480	500	50	8	[0, 41, 58, 74, 77]	**6**
C	RaceHorsesC	832**⋅**480	300	30	8	[0, 17]	**2**
D	BasketballPass	416**⋅**240	500	50	8	[0, 32, 56]	**4**
D	BlowingBubbles	416**⋅**240	500	50	8	[0, 41, 77]	**3**
D	RaceHorsesD	416**⋅**240	300	30	8	[0, 17]	**2**
E	KristenAndSara	1280**⋅**720	600	60	8	[0, 63, 67]	**3**
E	Johnny	1280**⋅**720	600	60	8	[0, 27, 63]	**3**
E	FourPeople	1280**⋅**720	600	30	8	[0, 41, 56, 58]	**4**

**Table 2:** List of object class IDs from the prepared ground truths adapted from [1].

Class ID	Object class name	Class ID	Object class name
0	person	41	cup
1	bicycle	56	chair
13	bench	58	potted plant
17	horse	63	laptop
26	handbag	67	cell phone
27	tie	74	clock
32	sports ball	77	teddy bear

Similarly, relative bounding box width and height, w and h, are obtained from the absolute width and height, $w^{*}$ and $h^{*}$, as:

(3) $$w=\frac{w^{*}}{N}$$

(4) $$h=\frac{h^{*}}{M}$$

The conversion between relative coordinates and absolute coordinates is also explained in [1].

**Fig. 1:**
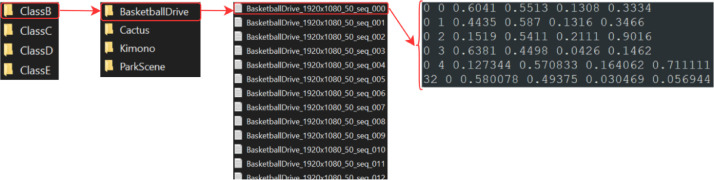
Ground truth of sequence BasketballDrive and the corresponding file structure.

**Fig. 2:**
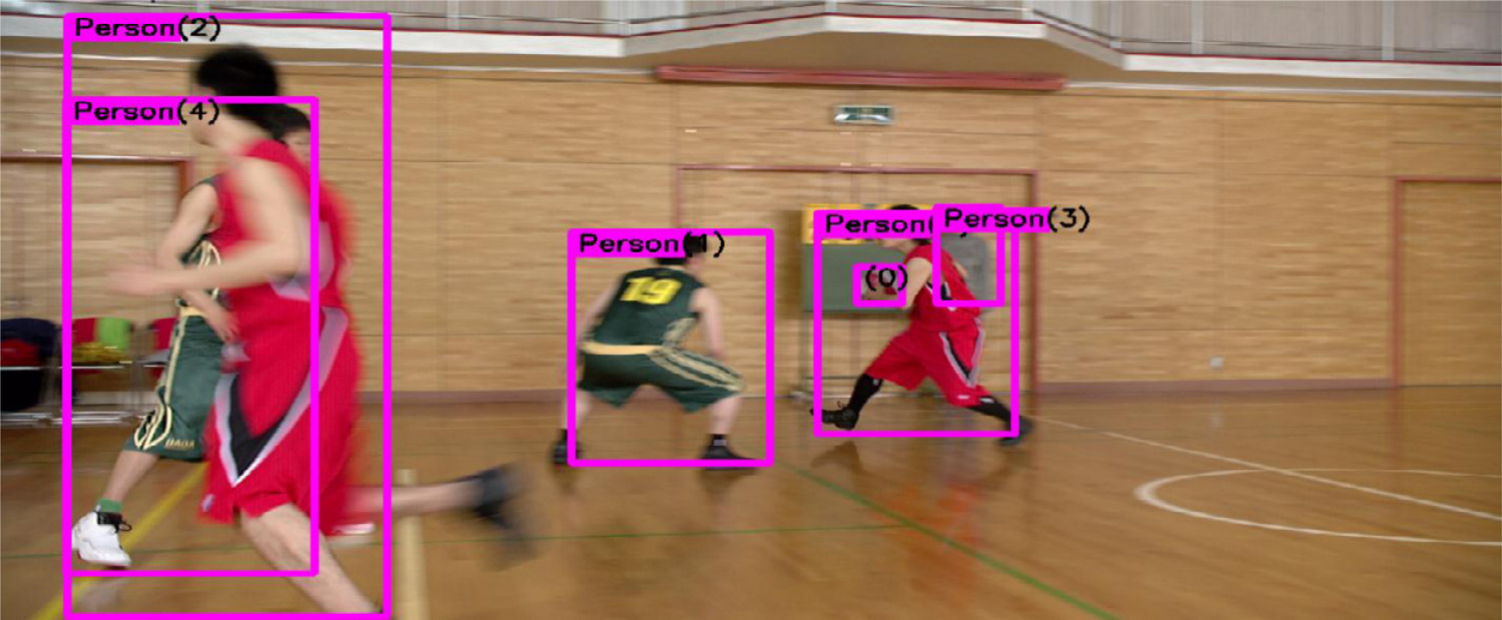
Visualized annotations on the sequence BasketballDrive at frame 0.

The folder and file structure of SFU-HW-Tracks-v1 is illustrated on the example of the BasketballDrive sequence in Fig. 1. The corresponding annotations can be visualized overlaid on the image frame using YOLO Mark^1^ [4], as shown in Fig. 2. In the first frame in this sequence, there are four objects from the “person” class (class ID 0) with object IDs from 0 to 3. There is also a single “sports ball” object (class ID 32) with object ID 0. The combination of class ID and object ID uniquely identifies each annotated object.

## 2 Experimental Design, Materials and Methods

Tracking annotations in SFU-HW-Tracks-v1 were created based on object detection annotations in SFU-HW-Objects-v1 [1], which contain the following information for each object: [Class ID, x*,* y*,* w*,* h]. However, SFU-HW-Objects-v1 is not suitable for tracking purposes because there is no annotation distinguishing different objects from the same class. Therefore, we further created unique object IDs within each class, which enables distinction of different objects in each class. Further, the same object ID is used for the same object in different frames, which allows computing tracking metrics. These object IDs are included in the second column of the provided annotation files.

We used normalized cross-correlation (NCC) [5] to measure the similarity between two bounding boxes, where each contains an object. To find matching locations for objects in neighboring frames (n and n+1), we computed NCC for all possible pairs of object bounding boxes between these two frames. For each object bounding box in frame n, we take as its best match the box in frame n+1 that gives the highest NCC score. If the NCC score is greater than the threshold value (0.6 in most sequences), we copy the corresponding object ID from frame n to the best-matched box in frame n+1. If the NCC score of the best-matched box is less than the threshold value, we manually assign an object ID to that object in frame n+1 via visual inspection. The threshold value was manually adjusted in the range [0.55, 0.75] in several sequences to account for different characteristics of objects and their appearance.

**Fig. 3:**
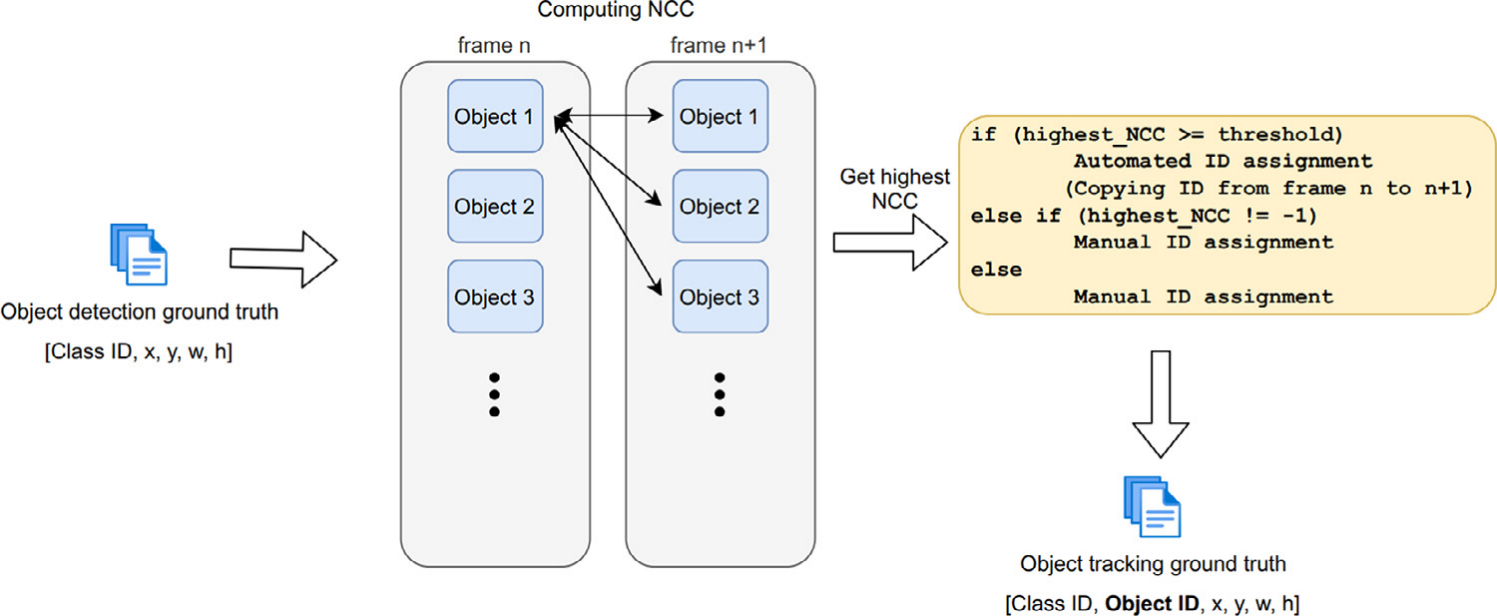
Semi-automated annotation process of object ID assignment adapted from [6].

If a particular object does not exist in frame n but exists in frame n+1 (i.e., the object has entered the scene), we defined NCC score as −1. In this case, we manually assign an object ID for the object in frame n+1. Such a situation could occur when an object disappears and re-appears due to occlusion, or appears for the first time. The manual ID assignment was conducted by visualizing the annotations on the frame with YOLO Mark, comparing the bounding boxes, and/or using the object annotation files. After assigning an ID, the annotation process proceeds with the next frame. Fig. 3 summarizes the semi-automated process of annotating object IDs.

## Ethics Statements

No human, animal subjects, and data from social media platforms were involved in this work.

## CRediT Author Statement

**Takehiro Tanaka**: Methodology, Software, Data curation, Writing - Original draft, Visualization. **Hyomin Choi**: Conceptualization, Methodology, Software, Data curation, Supervision, Writing - Review & Editing. **Ivan V. Bajić**: Conceptualization, Supervision, Writing - Review & Editing, Project administration, Funding acquisition.

## Declaration of Competing Interest

The authors declare that they have no known competing financial interests or personal relationships that could have appeared to influence the work reported in this paper.

## Footnotes

1 We slightly modified YOLO Mark to show the object ID for each object.

## References

[1] Choi H., Hosseini E., Alvar S.R., Cohen R.A., Bajić I.V. (2021). A dataset of labelled objects on raw video sequences. Data in Brief.

[2] Bossen F. (2013). Common test conditions and software reference configurations. JCTVC-L1100.

[3] Lin T.-.Y. (2014). Microsoft COCO: common Objects in Context,” in *Computer Vision – ECCV 2014*. Cham.

[4] Alexey, (2021). https://github.com/AlexeyAB/Yolo_mark.

[5] Zhao F., Huang Q., Gao W. (May 2006). Image matching by normalized cross-correlation. Proc. *IEEE ICASSP*.

[6] Tanaka T. (2021).

